# Folate Deficiency and Medication-Induced Severe Pancytopenia in a Bilateral Lung Transplantee

**DOI:** 10.7759/cureus.65780

**Published:** 2024-07-30

**Authors:** Nathan M Au-Yeung, Nicholas S Regennitter, Jacob Stepherson, Justin Seele, Ethan Rosenblatt, William Carter

**Affiliations:** 1 Hospital Medicine, Ochsner Clinic Foundation, New Orleans, USA; 2 Anesthesiology, Ochsner Clinic Foundation, New Orleans, USA

**Keywords:** pancytopenia, drug-induced pancytopenia, folate deficiency, lung transplant, transplant recipient, severe pancytopenia, bilateral orthotropic lung transplant

## Abstract

Folate is a water-soluble vitamin that is essential to DNA synthesis and replication. Its deficiency is a leading cause of megaloblastic anemia, which is often asymptomatic but can present with nonspecific symptoms, such as fatigue and lightheadedness. Folate deficiency can rarely present with pancytopenia, which has been described in past case reports but even more scarcely in transplant recipients. We present a 74-year-old bilateral lung transplantee who presented with presyncope and was found to have severe pancytopenia with folate deficiency during the initial workup. Some medications, including mycophenolate mofetil, valganciclovir, and posaconazole were held. Peripheral blood smear showed blastoid cells, but follow-up imaging and flow cytometry negated any concern for a malignant process. Bone marrow biopsy showed an extremely hypocellular marrow with marked trilineage hypoplasia. He required blood product transfusions, but his admission was overall uneventful with no life-threatening sequelae. His blood counts improved with folate replacement and discontinuation of offending medications. He was discharged after nine days in stable condition. Two months later, he experienced a milder and self-limited recurrence of pancytopenia with normal folate and cobalamin levels.

## Introduction

Folate (vitamin B9) is a water-soluble vitamin present in various vegetables, fruits, and meats that is vital for proper DNA synthesis and replication along with cobalamin (vitamin B12). Folate and cobalamin deficiencies are leading causes of megaloblastic anemia, a category of anemias characterized by the presence of large abnormally developed red blood cell (RBC) precursors called megaloblasts that result from impaired DNA synthesis. Folate deficiency is often asymptomatic and discovered incidentally on routine testing, but it can manifest nonspecifically as fatigue, shortness of breath, tachycardia, lightheadedness, and pallor, particularly in more severely anemic patients. Etiologies vary widely and include alcohol abuse, malnutrition, malabsorption, antiepileptics, and antineoplastic agents [1]. Pancytopenia is a rarer consequence of folate deficiency, with scattered reports describing pancytopenia of varying severities resulting from folate and/or cobalamin deficiencies in various patients, including pregnant women, alcoholics, and adolescents [2-4]. Reports of pancytopenia associated with megaloblastic anemia in transplant patients are even rarer, with a reported case in a renal transplant recipient who did not have folate or cobalamin deficiency [5]. Evaluation includes a complete blood count with differential; peripheral blood smear; reticulocyte count; levels of folate, cobalamin, homocysteine, and methylmalonic acid; and additional workup for specific etiologies as indicated [1]. We present a case of an elderly man with a bilateral lung transplant who initially presented with presyncope and was found to have severe pancytopenia and folate deficiency during his admission.

## Case presentation

A 74-year-old male presented to the emergency department with his wife for an episode of presyncope. He became minimally responsive while walking and required assistance to sit down. He reported dyspnea on exertion for 2.5 months. He also noticed worsening of preexisting oral ulcers (with decreased oral intake for one week) and new bilateral upper extremity hematomas since starting apixaban for bilateral deep vein thromboses and atrial fibrillation one month ago. His oral ulcers and pancytopenia first developed three months prior; at the time, his ulcers temporarily resolved but then quickly returned. He denied true loss of consciousness, fever, trauma, and other symptoms. His medications included azithromycin, posaconazole, valganciclovir, and atovaquone for antimicrobial prophylaxis and prednisone 5 mg, tacrolimus 0.75 mg, and mycophenolate mofetil (MMF) 360 mg twice daily for immunosuppression. He denied any history of smoking or illicit drug use and consumes six cans of beer per week.

Regarding previous medical history, he received a bilateral lung transplant for idiopathic pulmonary fibrosis four years prior. His lung donor was positive for Epstein-Barr virus (EBV) and cytomegalovirus (CMV). Human leukocyte antigen typing was not recorded. His postoperative recovery was complicated by atrial fibrillation with rapid ventricular response, pericardial and pleural effusions, *Mycobacterium stephanolepidis* pneumonia, and acute transplant rejection within the next five months. He had a subsequent routine and uneventful follow-up up to two months prior to our case when he was admitted for dehydration, worsening oral ulcerations, several episodes of epistaxis and hemoptysis, early satiety, generalized weakness, and 17-lb weight loss from limited oral intake. Workup revealed acute on chronic anemia, thrombocytopenia of 50 K/µL, and mild leukocytosis; the latter two resolved by discharge. Imaging revealed a right lower lobe pneumonia treated with piperacillin-tazobactam.

**Figure 1:**
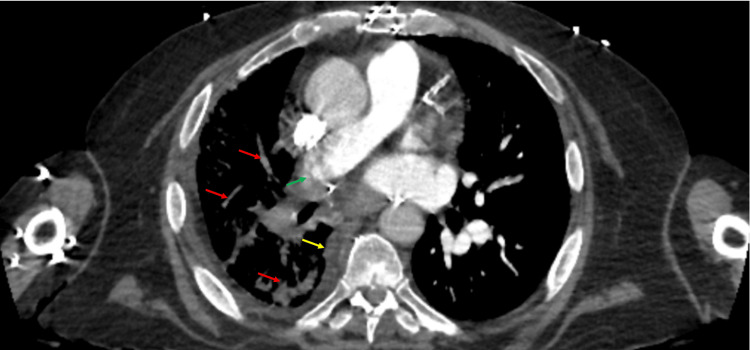
Computed tomography scan of the chest with contrast demonstrating reticular and patchy consolidative opacities in the right lung (red arrows), pleural thickening at the right lung base (yellow arrow), and high-grade stenosis in the distal right main pulmonary artery (green arrow).

In our emergency department, the patient was afebrile, tachycardic, normotensive, and in no respiratory distress. Initial workup revealed a hemoglobin level of 6.5 g/dL, mean corpuscular volume (MCV) of 118 fL, platelet count of 1 K/µL, white blood cell (WBC) count of 0.49 K/µL, and absolute neutrophil count of 144 cells/µL (Table 1). The corrected reticulocyte count was low at 0.2%. Chest x-ray showed bilateral patchy airspace and ground-glass opacities in the right lung. A computed tomography scan of the chest showed extensive reticular and patchy consolidative opacification throughout the right lung with pleural thickening and high-grade stenosis of the right pulmonary artery (Figure 1), whereas a scan of the head showed no acute abnormality. His apixaban, aspirin, MMF, and valganciclovir were held. He was started on vancomycin and cefepime for empiric neutropenic coverage, transfused one unit of packed RBCs and platelets, and admitted for further evaluation and management.

**Table 1:** Blood counts throughout admission Hct: hematocrit; Hgb: hemoglobin; Plt: platelets; WBC: white blood cells

Component	Reference values	On admit	Day 1	Day 2	Day 3	Day 4	Day 5	Day 6	Day 7	Day 8	Day 9
Hgb (g/dL)	14-18	6.5	6.9	7.8	7.7	7.7	7.6	7.6	7.6	8.1	7.6
Hct (%)	40-54	18.9	20.4	22.2	22.7	23.1	22.5	22.6	22.6	23.8	22.9
Plt (K/µL)	150-450	1	23	14	12	8	23	19	23	30	43
WBC (K/µL)	3.9-12.7	0.49	0.28	0.23	0.32	0.47	0.57	0.80	0.95	1.70	2.84

Further testing revealed a low folate level of 3.3 ng/mL (normal range 4-24 ng/mL) for which he began supplementation on day 1. The cobalamin level was normal. Fungal serology and viral DNA were negative, though he did have elevated levels of IgG antibodies to EBV nuclear and viral capsid antigens (Table 2). Previous serology was positive only for IgG antibodies against CMV and EBV capsid and nuclear antigens, but past records did not include a quantitative level. Iron studies showed elevated iron at 214 µg/dL (normal range 45-160), saturated iron at 84% (20-50), and ferritin at 1,976 ng/mL (20-300) as well as decreased transferrin at 173 mg/dL (200-375) and normal total iron binding capacity at 256 µg/dL (250-450). ADAMTS13 activity was normal. Erythropoietin (EPO) and lactate dehydrogenase (LDH) were significantly elevated at 1,230 mIU/mL (normal range 2.6-18.5) and 325 U/L (110-260), respectively. Fibrinogen was initially elevated but decreased to normal limits (182-400 mg/dL) by day 2. Haptoglobin levels were initially normal but began decreasing on day 3 to <10 mg/dL (30-250) by discharge. His total bilirubin reached a maximum of 1.8 mg/dL (0.1-1) but decreased to 1.1 by discharge. He tested negative on three indirect Coombs tests during and after admission.

**Table 2:** Infectious serology Ab: antibody; Ag: antigen; CMV: cytomegalovirus; EBV: Epstein-Barr virus; HAV: hepatitis A virus; HBV: hepatitis B virus; HbsAg: hepatitis B surface antigen; HCV: hepatitis C virus; HIV: human immunodeficiency virus; N/A: not available; VCA: viral capsular antigen, SARS-CoV-2: severe acute respiratory syndrome coronavirus 2. Asterisk denotes elevated values.

Test	Result	Ref value
Aspergillus antigen (index value)	<0.5	<0.5
Blastomyces Ag	Not detected	N/A
CMV DNA (IU/mL)	Not detected	<50
Coccidioides Ab	Negative	N/A
Cryptococcal antigen	Negative	N/A
EBV DNA (IU/mL)	Not detected	<12
EBV early antigen IgG Ab (U/mL)	<5	<9
EBV nuclear Ag IgG Ab (U/mL)	152*	<18
EBV VCA IgG Ab (U/mL)	431*	<18
EBV VCA IgM Ab (U/mL)	13.4	<36
Fungitell assay (pg/mL)	<31	<60
HAV IgM Ab	Non-reactive	N/A
HBV IgM Ab	Non-reactive	N/A
HBsAg	Non-reactive	N/A
HCV IgM Ab	Non-reactive	N/A
Histoplasma urine Ag	Not detected	N/A
HIV 1/2 Ag/Ab	Non-reactive	N/A
Parvovirus B19 IgG Ab	Positive	N/A
Parvovirus B19 IgM Ab	Negative	N/A
SARS-CoV-2 RNA	Negative	N/A

**Figure 2:**
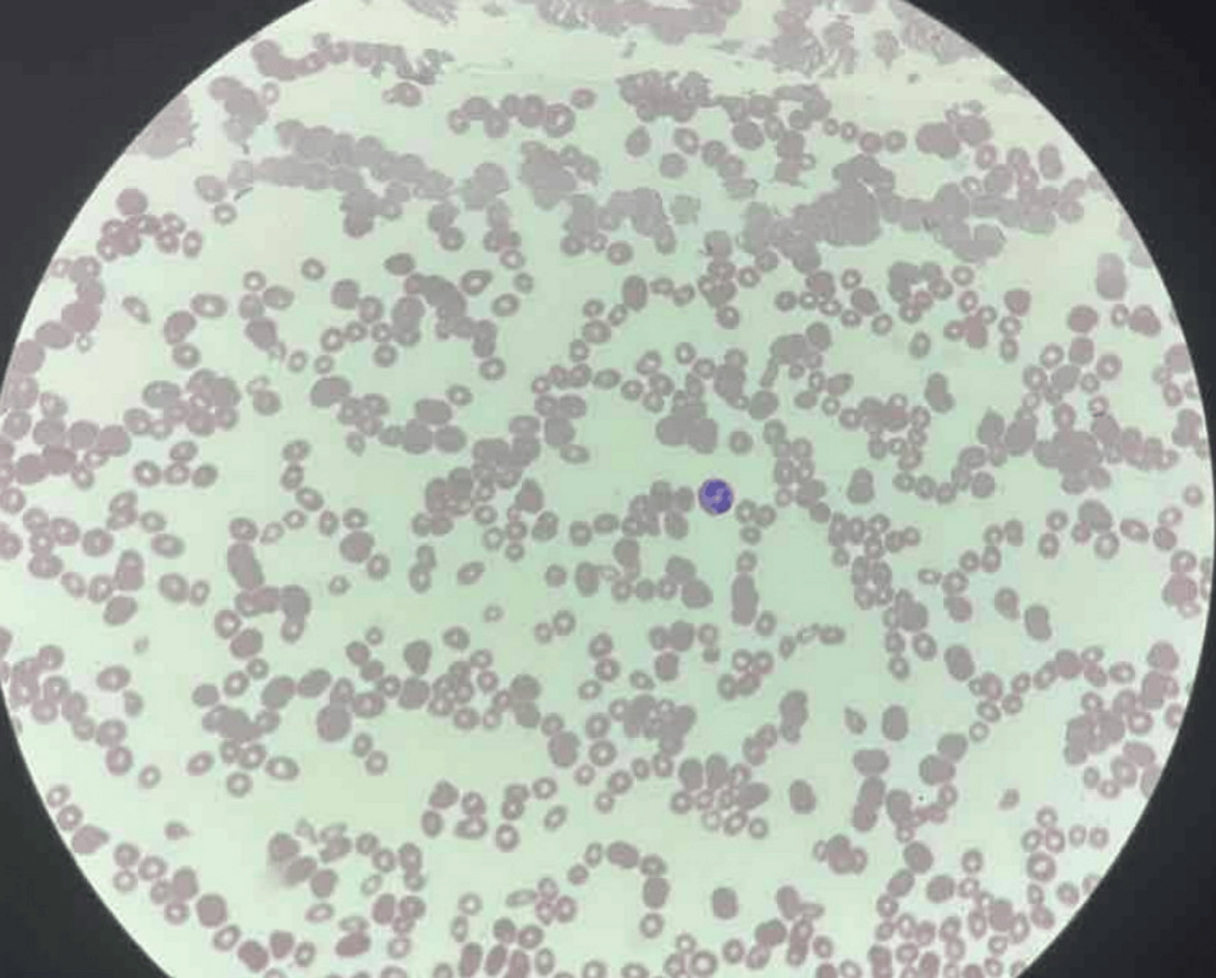
A peripheral blood smear showing severe pancytopenia with moderate anisopoikilocytosis. No blastoid cells are seen in this section.

On admission, he was empirically treated with dexamethasone 40 mg out of concern for immune thrombocytopenia (ITP), though this was deemed unlikely as involvement of other blood cell lineages was suggestive of a broader hematologic disease process. A blood smear (Figure 2) obtained on admission and read one day after showed blastoid cells, concerning acute leukemia. At this point, dexamethasone was discontinued after two doses and the patient switched back to his home prednisone due to a greater concern for leukemia instead of ITP. Subsequent flow cytometry showed no supporting evidence. Follow-up imaging showed no osseous lesion. Bone marrow (BM) biopsy was obtained on day 2.

**Figure 3:**
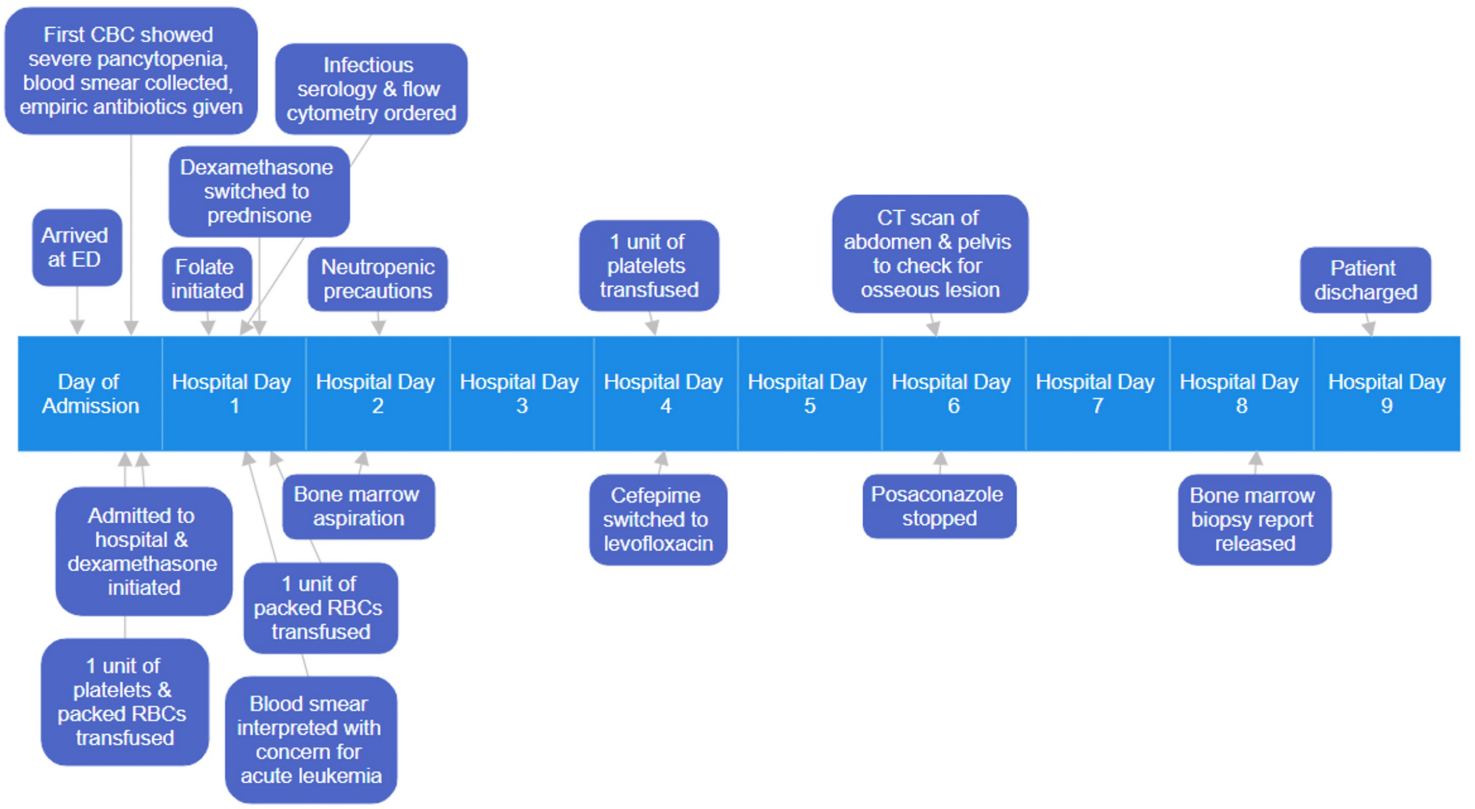
Events throughout admission CBC: complete blood count; CT: computed tomography; ED: emergency department; RBC: red blood cell

Otorhinolaryngology was consulted on day 2 and recommended dexamethasone rinse and magic mouthwash, which provided some relief for his oral ulcers. WBC count dropped to 0.24 K/µL the same day, necessitating neutropenic precautions, but slowly recovered after. Platelet count increased to 23 K/µL after transfusion but decreased to 8 K/µL on day 4, requiring another transfusion, after which it also slowly recovered. He required another unit of packed RBCs on day 1, though his hemoglobin and hematocrit remained stable after. Because he showed no signs suggesting infection, his antibiotics were switched to levofloxacin for neutropenia prophylaxis on day 4. Posaconazole was stopped on day 6 due to concern about interaction with his immunosuppressants. He continued the rest of his admission (Figure 3) with no signs of fever, hypoxia, cough, or worsening dyspnea and was hemodynamically stable throughout. His BM biopsy report was finalized one day before discharge and showed an extremely hypocellular marrow (5%-10% total cellularity) and marked trilineage hypoplasia without increased blasts. After nine days with improved blood counts, the patient was discharged with instructions to stop taking MMF, posaconazole, and valganciclovir, begin indefinite folate supplementation, and increase his tacrolimus dose from 0.75 to 1 mg. Levofloxacin was discontinued on discharge when his neutropenia resolved. Six days later, the WBC count improved to 7.29 K/µL and the platelet count improved to 161 K/µL.

During outpatient follow-up, he resumed his MMF at 180 mg twice daily. His outpatient hematologist and transplant pulmonologist noted that his pancytopenia was likely due to medication side effects and folate deficiency, and it improved with the discontinuation of posaconazole and initiation of folate supplementation. His oral ulcers were fully resolved without further issues. About two months after discharge, he experienced another decrease in platelet count to 31 K/µL and WBC count to 2.78 K/µL. Hemoglobin was stable at 9 g/dL. Folate, cobalamin, and tacrolimus levels remained normal. He was instructed to hold apixaban but continue MMF. Both counts spontaneously recovered over the next three weeks.

## Discussion

Our patient presents an interesting case because while he had a mild folate deficiency, his pancytopenia was disproportionately severe with a presenting platelet count of 1 K/µL and WBC count of 0.49 K/µL. There is a dearth of literature on pancytopenia associated with folate deficiency and megaloblastic anemia, as pancytopenia is an unusual side effect of folate deficiency in general, let alone in transplant patients. The circumstances of past reports also differ from our case. One describes a pregnant patient with a very low folate level (and slight cobalamin deficiency) yet milder thrombocytopenia and leukopenia [2], another describes three patients with alcohol abuse who presented with low to undetectable folate levels yet milder leukopenia and thrombocytopenia [3], a third describes a 15-year-old female patient with a history of anorexia who presented with both folate and cobalamin deficiency but milder pancytopenia [4], and a fourth describes a renal transplantee who suffered less severe pancytopenia from azathioprine-induced megaloblastic anemia with normal folate and cobalamin levels [5]. Our report describes a case of a lung transplantee, which to our knowledge has not been reported in the current literature.

Solid organ transplant patients may be susceptible to folate deficiency or other anemias for various reasons. Drugs such as azathioprine and MMF, which are used in long-term immunosuppression for transplant patients, can cause megaloblastic anemia via alteration of purine metabolism [6]. Other studies have observed folate deficiencies in cardiac and renal transplant recipients, and in a subset of patients (including lung allograft recipients), elevated homocysteine levels despite normal folate levels and absorption, suggesting that folate metabolism, activation, intracellular function, and/or excretion may be affected as well. These studies suggest various mechanisms, including deficiency of vitamin B6 (essential for homocysteine metabolism), abnormal renal function (also important in homocysteine metabolism as well as folate excretion and reabsorption), and poor dietary vitamin intake [7,8]. Unfortunately, our patient did not have any homocysteine or vitamin B6 level on record for further analysis. His family also reported no dietary abnormalities. A rare cause of folate deficiency is hereditary folate malabsorption, in which impaired intestinal folate absorption and transport into the central nervous system result in several symptoms, including megaloblastic anemia, immunodeficiency, failure to thrive, oral mucositis, developmental delay, and diarrhea. However, it is very rare (with less than 100 reported cases), and patients usually have a family history (ours did not have any), manifest symptoms in infancy, have significant neurologic symptoms (whereas he was at baseline mentation), and optimally respond to leucovorin [9].

In contrast, the literature exploring folate deficiency in lung transplants is very lacking. One study describes normocytic/macrocytic anemia predominantly as a result of low EPO levels instead (one patient was cobalamin deficient and all had normal folate levels) in lung transplant recipients [10]. While we suspect that folate deficiency contributed, the degree to which his pancytopenia was a consequence of his transplant, his specific personal health issues, or another unknown mechanism is unclear, especially considering the second milder and self-resolving pancytopenia with normal folate levels he had two months later while on folate supplementation, half of his prior dose of MMF, and no longer on other offending medications. Future research evaluating and comparing plasma levels of vitamins, compounds, and metabolites relevant to megaloblastic anemia and pancytopenia in lung transplantees could prove useful. In all transplant recipients, routine follow-up is vital because both their transplant and medications put them at risk for a variety of health consequences, as illustrated by our patient and the existing literature.

Our patient’s pancytopenia can also be attributed to medication side effects and interactions. Myelosuppression is a prominent side effect of MMF [11], and posaconazole is a known inhibitor of cytochrome P450 3A4 (CYP450) [12], which is one of several enzymes that metabolizes the active form of MMF into metabolites excreted in the urine. However, these metabolites comprise minute fractions of MMF [13]. Tacrolimus is also metabolized by CYP450 enzymes [14], though associated hematologic toxicity is rare and usually presents as reversible pure red cell aplasia [15]. Tacrolimus-induced pancytopenia is even rarer, with two documented cases milder in severity compared to our patient [16,17]. Because our patient had recovered from his blood counts with tacrolimus at therapeutic levels throughout admission, it is unlikely that tacrolimus toxicity was the cause. Posaconazole is generally well-tolerated, and adverse effects include gastrointestinal complaints and hepatotoxicity, though hematologic toxicity has not been reported [18,19]. Unlike MMF and tacrolimus, valganciclovir is not significantly metabolized by CYP450 enzymes; it is instead rapidly metabolized in the intestinal wall and liver into ganciclovir, which is excreted by the kidneys without further change [20]. Severe pancytopenia secondary to valganciclovir has also been reported in four cases of patients with renal impairment on standard doses of valganciclovir (900 mg daily), all of whom also had significant BM hypocellularity [21]. Compared to these four patients, while our patient had a history of chronic kidney disease, his creatinine remained within normal limits and, along with his blood urea nitrogen, was nowhere near as elevated. However, it is plausible that his medications and vitamin deficiency may have had an additive or synergistic effect that ultimately resulted in pancytopenia that was just as severe.

Our patient’s laboratory results provide further insight into our hypothesis. An MCV >115 fL is more specific for folate or cobalamin deficiency than other causes of macrocytosis [1]. His iron studies are suggestive of iron overload, which could be explained by folate deficiency preventing the proper utilization of iron for new hemoglobin and RBC synthesis. This is further supported by his ferritin returning to normal limits after discharge. A previous report has also described iron overload in two folate-deficient patients [22], and our patient did not have any risk factors to suggest another etiology such as chronic liver disease (denied excessive alcohol intake, had a temporarily mildly elevated alanine aminotransferase, and had no significant hepatic abnormality on imaging), secondary iron overload (has never required transfusions before), or hemochromatosis (no characteristic physical findings or family history) [23]. Normal ADAMTS13 activity suggests that thrombotic thrombocytopenic purpura is an unlikely cause of his anemia and thrombocytopenia, especially considering that he did not exhibit other associated symptoms such as fever, abdominal pain, and altered mental status [24]. Elevated EPO and LDH can indicate hemolytic anemia, but this is unlikely due to a lack of compensatory reticulocytosis, positive indirect Coombs test, and acute hemoglobin decrease [25]. Elevated EPO and LDH could also be attributed to a pulmonary disease process stemming from his transplant (as evidenced by chest imaging) that has produced a chronic hypoxia causing tissue damage (and release of LDH) and compensatory EPO production; this could explain his elevated fibrinogen as inflammation enhances fibrinogen synthesis [26]. Our patient’s reticulocytopenia suggests BM failure as one would expect a reticulocytosis, especially in response to a hemoglobin below the transfusion threshold (<7 g/dL at our institution). Decreased haptoglobin is also a sign of hemolysis [27], but in our patient, it is more likely due to consumption from binding free hemoglobin from his resolving upper extremity hematomas.

In a transplant patient on long-term immunosuppression, it is important to exclude other causes of BM failure. Although not the most common consequences, pancytopenia, BM failure, and aplastic anemia (AA) have been observed in patients infected with parvovirus B19, human immunodeficiency virus (HIV), EBV, and CMV [28-31]. Fortunately, our patient had a negative serology for HIV and CMV. Although he was positive for parvovirus IgG antibodies, he was negative for IgM antibodies, indicating no recent infection. He did have elevated levels of IgG antibodies to EBV nuclear and viral capsid antigens, though this could be explained by his lung donor’s seropositivity for EBV. More recently, reports of pancytopenia following SARS-CoV-2 have emerged [32], though our patient tested negative. A viral etiology seems unlikely given that he had no symptoms suggestive of a viral syndrome, had no tests indicative of an acute infection, responded appropriately to conservative measures, and did not need any antiviral therapy. While AA can also cause severe pancytopenia and result in a markedly hypocellular marrow (as seen in our patient), it is a diagnosis of exclusion, and resolution of our patient’s pancytopenia after folate replacement, withholding offending medications, and no additional therapy would not be expected as most cases of AA require immunosuppressants, typically cyclosporine and anti-thymocyte globulin [33].

## Conclusions

We report a case of a 74-year-old male with a bilateral lung transplant who presented with presyncope and was found to have severe pancytopenia during the initial evaluation. There was initial concern for leukemia due to blastoid cells on the peripheral blood smear, but subsequent workup provided no evidence for a malignant process. BM biopsy showed an extremely hypocellular marrow with trilineage hypoplasia but no blasts. His admission was otherwise uneventful with no life-threatening sequelae. He improved after folate supplementation and discontinuation of offending medications. He was discharged after nine days with improved blood counts. Literature is scarce on pancytopenia secondary to folate deficiency in patients with solid organ transplants, especially lung transplants. We aimed to review the current literature and provide insight into the various factors, including medications and laboratory results, that should be considered when managing a transplant patient with severe pancytopenia.
